# 

**Published:** 1822-03

**Authors:** 


### MONTHLY CATALOGUE OF MEDICAL BOOKS.

An Essay on the Symptoms and History of Diseases, considered chiefly in their Relations to Diagnosis. By Marshall Hall, M.D. F.R.S. 6s.

Essays on Surgery and Midwifery; with Practical Observations and select Cases. By James Barlow, Surgeon. With Plates. 12s.

Conversations on Chemistry; in which the Elements of that Science are familiarly explained and illustrated by Experiments. In 2 vols. Eighth Edition, revised, corrected, and enlarged. 14s.

Shaw's Manual for the Student of Anatomy. Second Edition. 8vo. 12s.

Rigby's Essay on Uterine Hemorrhage. New Edition. 7s.

---

### NOTICES TO CORRESPONDENTS.

*We have received* Dr. EVANSON'S *Essay, and will notice it in our next Number. The subject deserves consideration.*

Mr. COOKE *will oblige us by informing the officers of the Hunterian Society, that we shall always be happy in promoting the views of that or any other scientific Institution, founded and conducted on liberal principles. We are thankful for his communication.*

Dr. DODS, *of Worcester, must really excuse us if we decline inserting his reply to our short notice of his "Physician's Guide." On looking back to that article, we cannot see any reason either for* Dr. DODS' *being dissatisfied with what we have said of his work, or for us to regret having discharged what we considered to be our duty. We beg to assure him that it is not for want of respect that we have decided on adopting this line of conduct.*

Mr. COLLIER, *of Norfolk street, Strand, is desirous that we should communicate to the profession a mode, which he proposes, for effectually rousing the system in cases of poisoning by narcotics. His method consists in scattering some of the* pubes dolichi prurientis *over the body of the patient, particularly about the head, neck, and arms. The effect is said to be almost immediate.*

*We beg to return our cordial thanks to* Dr. SILLAR *and* HOOD *for their excellent Communication.*

*We do not see any thing new in the Communication sent to us from the "Royal Jennerian Society," that requires to be noticed. When* Dr. WALKER *has any curious and important fact to communicate to the profession on the subject of Vaccination, we shall be happy to convey them to the public.*

